# Generation of complex human organoid models including vascular networks by incorporation of mesodermal progenitor cells

**DOI:** 10.1038/s41598-019-52204-7

**Published:** 2019-10-30

**Authors:** Philipp Wörsdörfer, Nahide Dalda, Anna Kern, Sarah Krüger, Nicole Wagner, Chee Keong Kwok, Erik Henke, Süleyman Ergün

**Affiliations:** Institute of Anatomy and Cell Biology, Koellikerstraße 6, University of Würzburg, 97070 Würzburg, Germany

**Keywords:** Developmental biology, Stem cells

## Abstract

Organoids derived from human pluripotent stem cells are interesting models to study mechanisms of morphogenesis and promising platforms for disease modeling and drug screening. However, they mostly remain incomplete as they lack stroma, tissue resident immune cells and in particular vasculature, which create important niches during development and disease. We propose, that the directed incorporation of mesodermal progenitor cells (MPCs) into organoids will overcome the aforementioned limitations. In order to demonstrate the feasibility of the method, we generated complex human tumor as well as neural organoids. We show that the formed blood vessels display a hierarchic organization and mural cells are assembled into the vessel wall. Moreover, we demonstrate a typical blood vessel ultrastructure including endothelial cell-cell junctions, a basement membrane as well as luminal caveolae and microvesicles. We observe a high plasticity in the endothelial network, which expands, while the organoids grow and is responsive to anti-angiogenic compounds and pro-angiogenic conditions such as hypoxia. We show that vessels within tumor organoids connect to host vessels following transplantation. Remarkably, MPCs also deliver Iba1^+^ cells that infiltrate the neural tissue in a microglia-like manner.

## Introduction

Organoids derived from human induced pluripotent stem cells (hiPSCs) are state of the art cell culture models to study mechanisms of development and disease^1,2^. The establishment of different tissue models such as intestinal^3^, liver^4^, cerebral^5^, kidney^6^ and lung organoids^7^ was published within the last years. These organoids recapitulate the development of epithelial structures in a fascinating manner. However, they remain incomplete as vasculature, stromal components and tissue resident immune cells are mostly lacking. All these cell types derive from mesenchymal tissue and it is well known that epithelial-mesenchymal interactions play a fundamental role during tissue development^8,9^.

Recent publications addressed this issue, especially with regard to organoid vascularization. Wimmer *et al*. demonstrated that human blood vessels self-organize and can be grown *in vitro*^10^. In order to vascularize cerebral organoids, Pham *et al*. added endothelial cells to the system^11^. But blood vessels are more complex than an endothelial tube. Larger vessels consist of multiple layers that contain cell types such as endothelial and smooth muscle cells, while even small capillaries rely on the support of pericytes and a basal lamina. Other groups generated vascularized neural organoids consisting of blood vessels and microglia^12,13^. However, in these cases, the heterologous vessels as well as microglia are host derived and invade the neural organoid after transplantation. A third study described that microglial cells innately develop within cerebral organoids *in vitro* due to contaminating mesodermal progenitors^14^. Nevertheless, formation of blood vessels was not detected.

Here we describe for the first time the specific integration of iPS cell-derived human mesodermal progenitors (MPCs) into organoids. We show that co-cultures or mixing of MPCs with either neural spheroids or tumor cells results in the formation of vascularized organoids *in vitro*. Moreover, the *in vitro* created blood vessels have the ability to connect to preexisting blood vessels of the chicken chorion allantois membrane (CAM) and might enable blood supply of implanted tissues. Besides providing a functional vasculature, we create mesenchymal-epithelial interfaces, an important developmental component during organogenesis.

## Results

**Figure 1:**
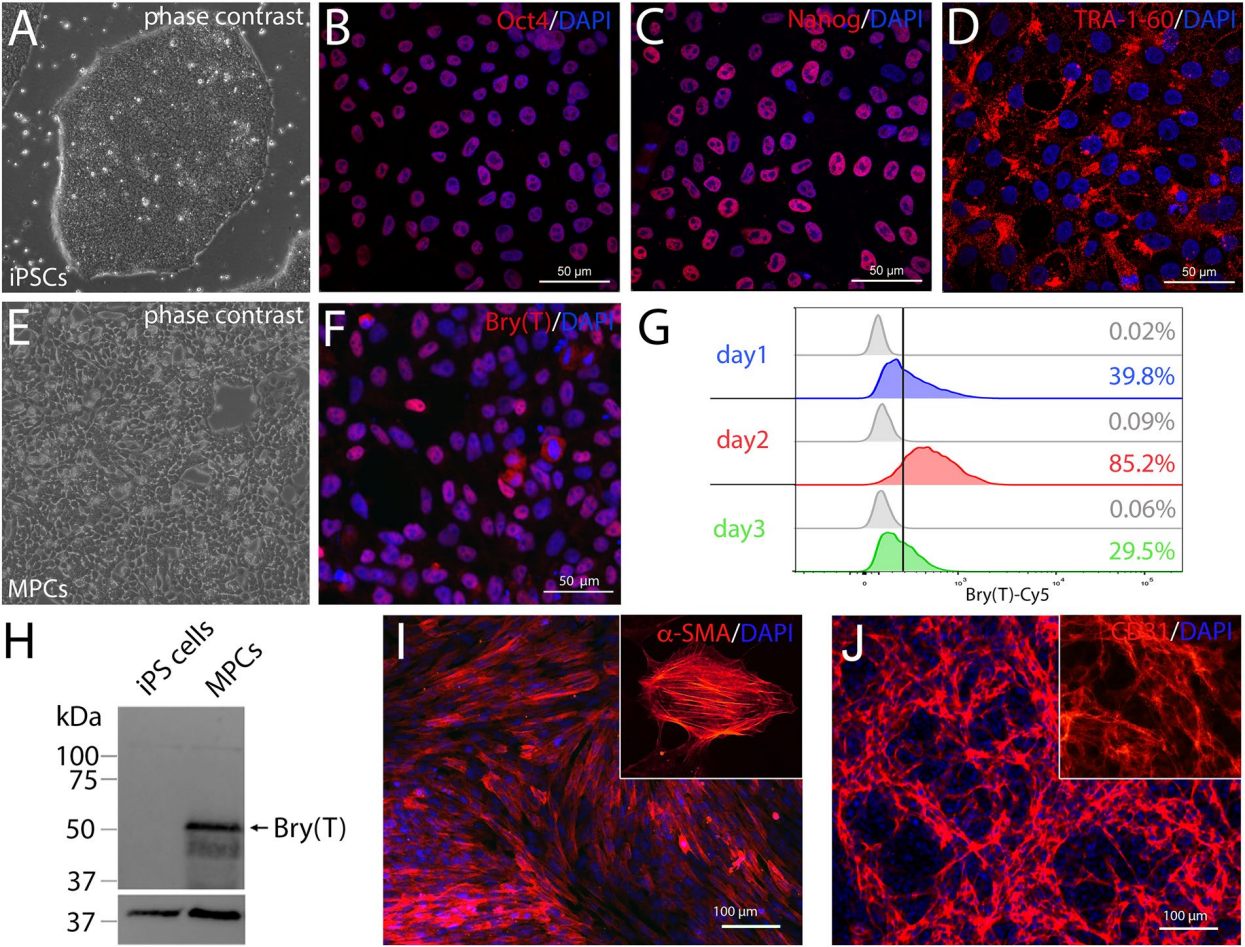
Induction of mesodermal progenitor cells (MPCs) from human iPSCs. (**A**) Human iPSCs display a typical morphology and grow as densely packed colonies. (**B–D**) iPSCs express the pluripotency markers Oct4, Nanog and Tra-1–60. (**E–F**) 3 days after mesodermal induction cellular morphology changed and the cells became Brachyury^+^ (Bry(T)). (**G**) At day 2 after induction 85% of the cells were Bry(T)^+^ as determined by flow cytometry. (**H**) Bry(T) expression was also demonstrated performing western blot analyses. As loading control β-Actin is detected. Full-length blots are presented in Fig. S1. (**J**–**K**) After treatment of MPCs with PDGF or VEGF for 10 days, the cells acquired a smooth muscle or endothelial cell fate, respectively.

**Figure 2:**
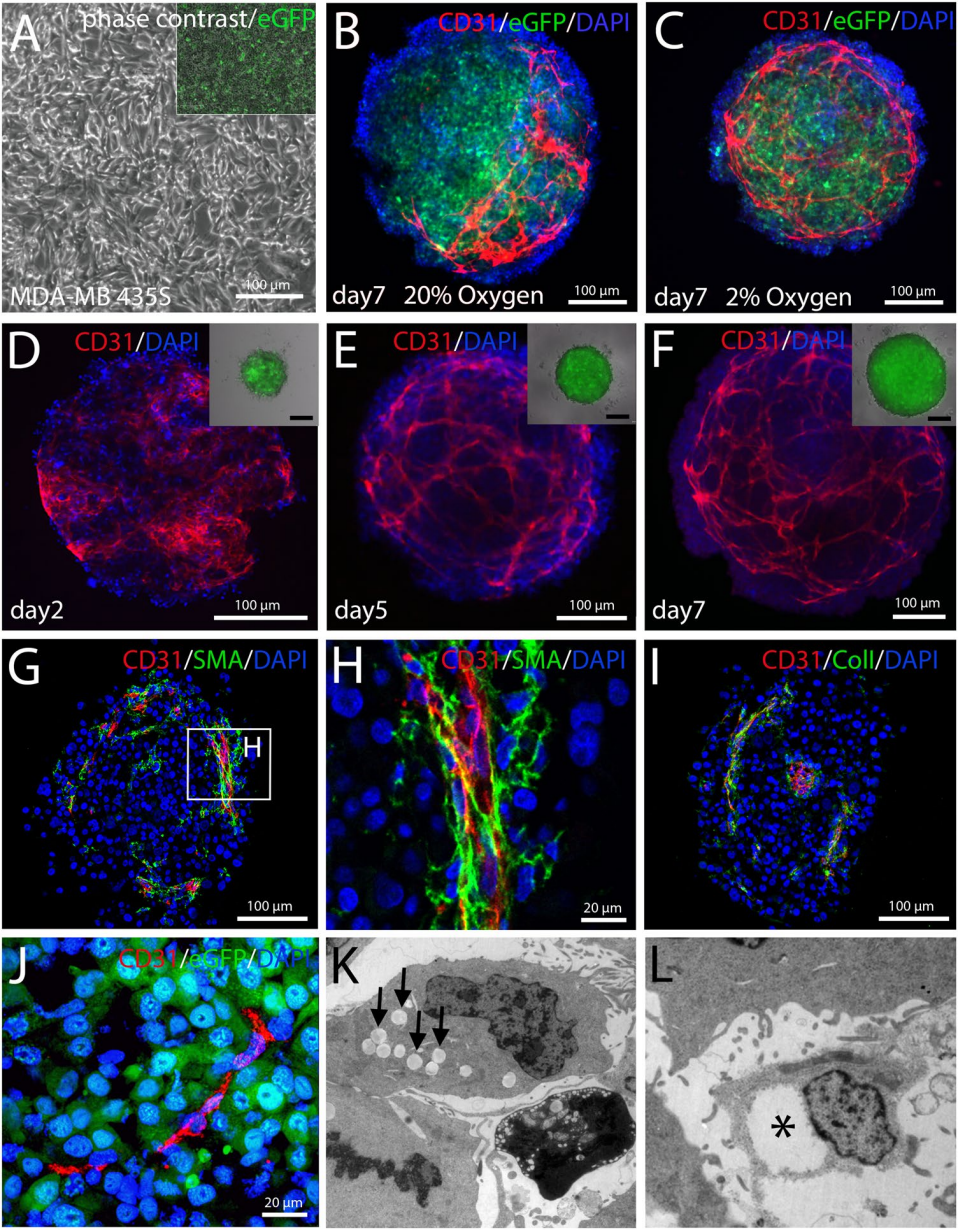
Generation and characterization of vascularized tumor organoids. (**A**) MPCs were mixed with GFP-labelled cells of the tumor cell line MDA-MB-435s in a 1:1 ratio (2000 cells each) and grown in suspension. (**B**) At 20% O_2_ endothelial cords were observed, arising only in one half of the aggregate. (**C**) Under hypoxic conditions an evenly distributed network of endothelial cords was observed. (**B–C**) Show aggregates at day 7 of culture. (**D**–**F**) CD31^+^ endothelial cells were observed as early as day 2 (**D**) and endothelial networks formed from day 4 on (**E**). The endothelial network expanded while tumor organoids increased in size (**F**). (**G**–**H**) CD31^+^ endothelial cords were enwrapped by SMA^+^ cells. (**I**) Moreover, a collagen type I^+^ extracellular matrix was found to accompany endothelial cords. (**J**) Endothelial sprouts directly penetrated the GFP^+^ tumor cell mass. (**K**–**L**) Early signs of lumen formation such as vacuole formation (**K**) and fusion (**L**) could be detected by transmission electron microscopy. (**B–F**) Show maximum intensity projections of whole mount stained cleared organoids. (**G–J**) Show stained paraffin sections.

**Figure 3:**
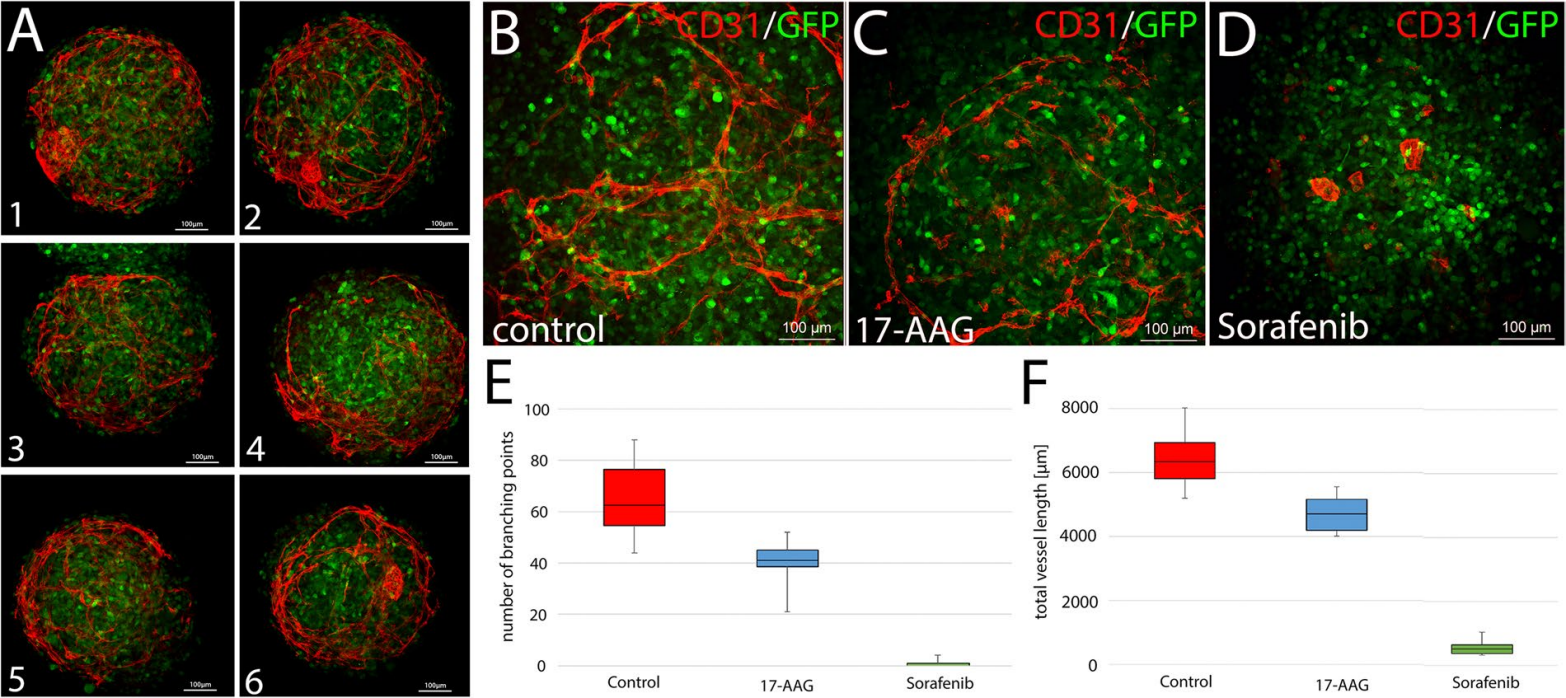
Treatment of vascularized tumor organoids with antiangiogenic drugs. (**A**) Production of vascularized tumor organoids was repeated several times yielding similar organoid sizes and vascular network distribution. Six randomly picked organoids from two independent experiments are depicted. (**B**–**D**) The single treatment of tumor organoids with 100 nM 17-AAG (**C**) or 100 nM Sorafenib (**D**) at culture day 4 results in a disturbed formation of endothelial networks compared to DMSO treated controls (**B**). Pictures were taken at day 8. Tumor cells express GFP. (**E**–**F**) Quantification of branching points (**E**) and total vessel length (**F**). For quantification randomly picked maximum intensity projections from 6 individual whole-mount stained organoids per condition were analyzed. 17-AAG and Sorafenib treated aggregates showed significantly fewer branching points and reduced vessel length (p < 0.0125 determined by Wilcoxon-Mann-Whitney test).

The aim of our study was to generate complex organoid models including stromal components, first of all blood vessels, but also fibroblasts and immune cells such as macrophages/microglia. These structures represent a microenvironment that creates important developmental niches. Embryologically, these cell types derive from the mesodermal lineage. Therefore, we induced Brachyury^+^ mesodermal progenitor cells (MPCs) from hiPSCs. This was achieved by activating Wnt signaling using the GSK3β-inhibitor Chir99021 and by adding BMP4^15^. We hypothesized that BMP4 signaling should favor a lateral plate mesodermal fate, similar to the situation in the embryo^16^. The lateral plate gives rise to the vascular system *via* blood islands which represent a source for both vascular wall and hematopoietic cells. During the initial 3 day-induction phase, hiPSCs completely lose pluripotency marker expression (Fig. 1A–D) and approximately 80% of the cells become positive for Brachyury at day 2 of differentiation (Figs 1E–H, S1). When MPCs are treated with either PDGF or VEGF, these cells differentiate into smooth muscle cells or endothelial cells, respectively (Fig. 1I,J), underscoring their mesodermal identity and their potential to produce the two major cell types of the blood vessel wall. In order to assess the vasculogenic potential of MPCs, we mixed them in a 1:1 ratio with green fluorescent protein (GFP)-labelled cells of the human tumor cell line MDA-MB-435s^17^ (Fig. 2A) and cultured the resulting aggregates in suspension. After 7 days we observed a vascular network that clustered mostly to one side of the tumor spheroid under normoxic conditions (20% O_2_) (Fig. 2B). However, after changing culture conditions to 2% O_2_, we found the network of capillary-like endothelial cords equally distributed within the organoid (Figs 2C, S2). Presumably, lowering the O_2_ concentration induces pro-angiogenic mechanisms, e.g. VEGF expression by the tumor cells *via* stabilization of hypoxia-induced factor (HIF1α), triggering endothelial cell proliferation and migration^18^. Under normoxic conditions, the vascular network forms only at one side of the aggregate, probably the side towards the bottom of the well, which might be exposed to lower oxygen concentrations. The observed vessel-like network surrounds a core of GFP^+^ tumor cells, but several CD31^+^ sprouts are also found directly within the tumor cell mass (Fig. 2J, Online Movie 1). While the aggregate grows from a diameter of 150 µm to approximately 500 µm, the endothelial network expands in a similar manner (Fig. 2D–F). The CD31^+^ endothelial cell cords are accompanied by α-smooth muscle actin (αSMA)^+^ cells indicating pericytes or smooth muscle cells being assembled into the vessel wall^19^ (Fig. 2G–H). Moreover, a collagen type I containing extracellular matrix is detected that is closely associated with the endothelial cells (Fig. 2I). Collagen type I is known to play an important role during endothelial cell migration and morphogenesis^20^. Some endothelial cells directly penetrate the GFP^+^ tumor cell core of the aggregates (Fig. 2J). Electron microscopy demonstrates vacuole formation (Fig. 2K) and fusion (Fig. 2L) within some cells of the tumor organoid suggesting lumen formation in parts of the capillary-like network^21^ (Fig. 2K–L). Production of vascularized tumor organoids was repeated several times yielding similar organoid sizes and vascular network distribution (Fig. 3A). To further address the aspect of reproducibility, we repeated the experiment with an additional iPS cell line (Sendai NHDF iPSC) yielding similar results (Figs S3, S4). The new iPS cell line was generated from commercially available dermal fibroblasts using a Sendai virus-based integration-free reprogramming method (Fig. S3). In addition, we randomly collected 20 organoids from one experiment and carefully quantified the organoid size and the surface area covered by CD31^+^ endothelial cells (Fig. S5A,B). We found that all tumor organoids display a similar CD31^+^ surface area (Fig. S5B). Regarding aggregate size, we observed a certain level of variation (500 µm^2^ +/− 71,5 µm^2^), probably due to pipetting errors (Fig. S5B). We did not detect any CD31^+^ cells in spheroids consisting of tumor cells only (Fig. S5A).

Next, we treated the tumor organoids with the tyrosine kinase inhibitor Sorafenib and the HSP90 inhibitor 17-AAG that had been shown to interfere with angiogenesis^22–24^. In both cases, we observe a disturbed formation of endothelial networks (Fig. 3B–F) demonstrating the suitability of the model for drug testing applications and responsiveness to anti-angiogenic compounds.

**Figure 4:**
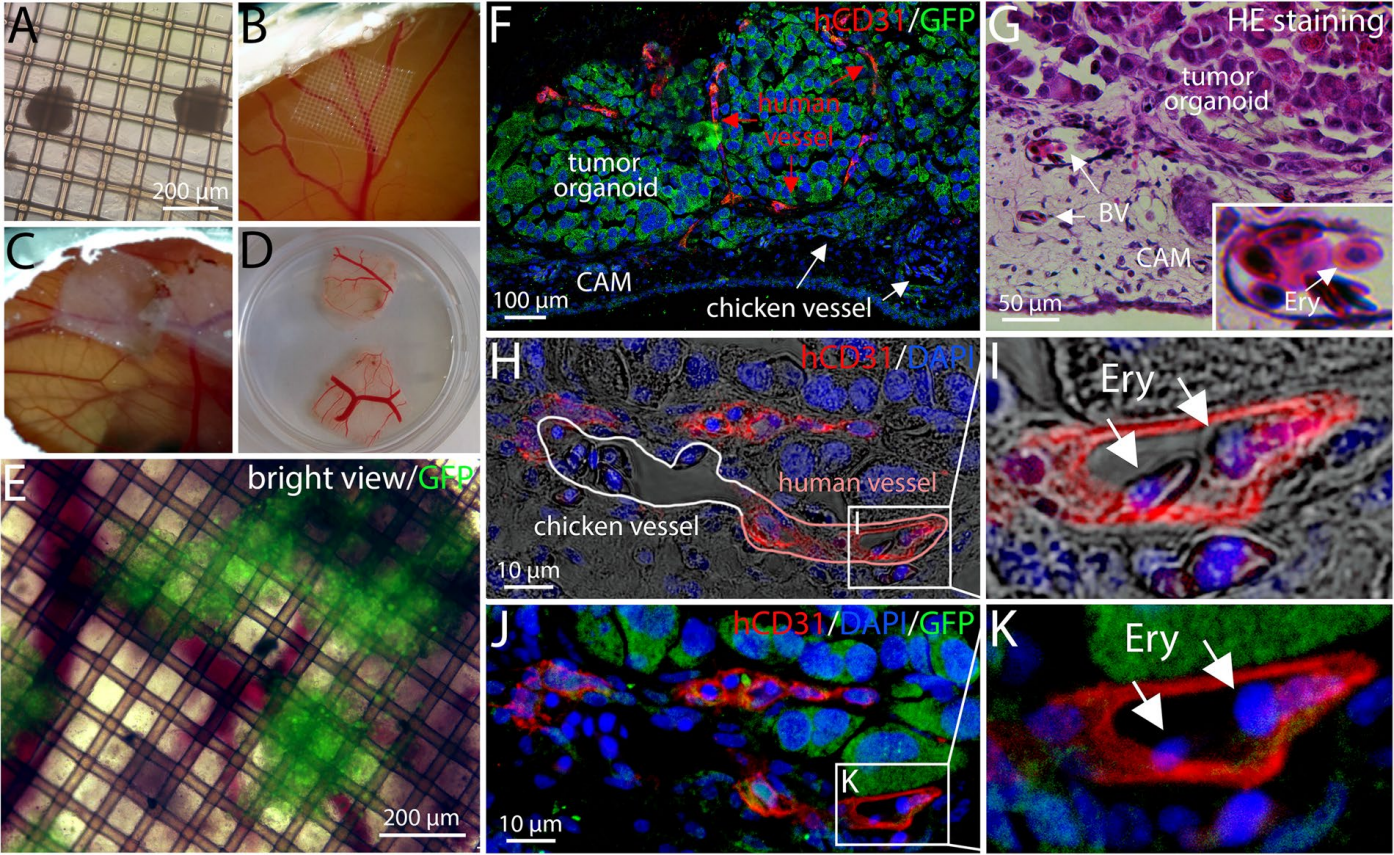
Transplantation of vascularized tumor organoids on the chicken chorion allantois membrane (CAM). (**A**) Tumor organoids were transferred into Matrigel and deposited on a nylon mesh. (**B**) The mesh was placed upside down on the CAM at ED 7. (**C**–**D**) After 3 days the mesh was removed. (**E**) GFP^+^ organoids could be observed in close neighbourhood to chicken vessels. (**F**) Human vessels were detected using a human-specific CD31 antibody. CD31^−^ chicken vessels can be also observed. Tumor cells are GFP^+^. (**G**) Blood vessels (BV) were found around the transplanted organoid. The inset shows a higher magnification of a blood vessel with luminal erythrocytes (Ery). (**H**) A CD31^−^ chicken vessel directly connected to a CD31^+^ human capillary is depicted. Blood cells can be found inside the chicken and the human vessel indicating a functional connection. (**I**) A higher magnification of H is shown. (**J**) The same picture detail as shown in H is depicted. Instead of phase contrast, the GFP^+^ tumor cells are shown. (**K**) A higher magnification of J is shown.

To test the functionality of the vascular network, we transplanted 4 days old tumor organoids on the CAM of a chicken embryo at ED 8. For that purpose, 10–20 tumor organoids were collected and transferred into 10 µl Matrigel. The gel and the organoids were transferred on a 1.5 cm^2^ nylon mesh (Fig. 4A). The mesh was placed upside down on the surface of the CAM for 3 days (Fig. 4B,C, Online Movie 2). When the mesh was cut out and removed (Fig. 4D), we found GFP^+^ tumor organoids in close neighbourhood to chicken blood vessels (Fig. 4E). We performed paraffin sections and observed CD31^+^ vascular structures within the GFP^+^ tumor tissue. To discriminate between human and chicken vessels, we used an antibody that detects human but not chicken CD31 (hCD31). Chicken blood cells were found within blood vessels around the tumor demonstrating blood perfusion (Fig. 4G). Moreover, we observed that human blood vessels directly connect to chicken vessels (Fig. 4H) and that chicken blood cells are detectable within the human vessels of the organoid (Fig. 4H–K, Fig. S6).

**Figure 5:**
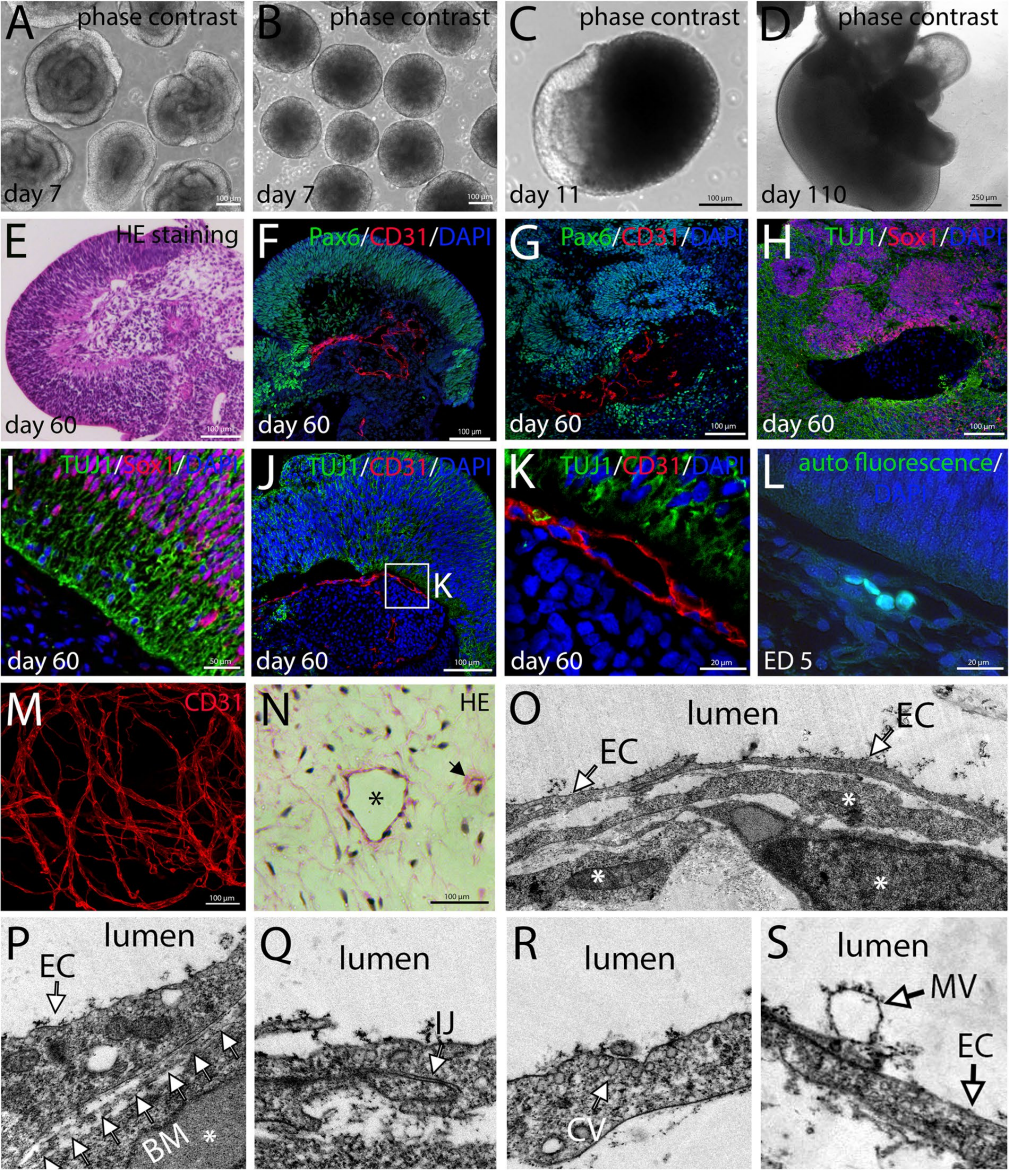
Characterization of vascularized neural organoids. Neural spheres consisting of Sox1^+^ neuroepithelial cells (**A**) and Brachyury^+^ mesodermal spheres. (**B**) Spheres were brought in co-culture. The formation of chimeric neuro-mesodermal aggregates was observed (**C**). The aggregates were cultured for up to 280 days (**D**). (**E**) HE-staining of sections showing the interface between neuroepithelial (left side) and mesenchymal part (right side). (**F**–**G**) Immunofluorescence analyses showing the Pax6^+^ neuroepithelium and CD31^+^ endothelial cells. (**H**–**D**) The neural part consists of Sox1^+^ stem cells and TUJ1^+^ neurons. (**J**–**K**) At the interface between neuroepithelium and mesenchyme, CD31^+^ vessels form a perineural plexus. (**L**) A capillary at the perineural plexus in an ED 5 chicken embryo is depicted. Blood cells show green autofluorescence. (**M**) Within the mesodermal part an extensive network of blood vessels can be observed. M shows a maximum intensity projection of a whole mount stained organoid. (**N**) HE staining of paraffin sections from the mesodermal part of an organoid. The picture shows a vessel structure with clear lumen (asterisk) and a small capillary (arrowhead). (**O**–**S**) Transmission electron microscopic pictures showing the endothelium of vessel structures within the organoids. *Periendothelial cells, EC: endothelial cells, BM: basement membrane, IJ: intercellular junction, CV: caveolae, MV: microvesicle.

The experiments involving tumor cells demonstrate the capacity of MPCs to vascularize tissue models. Next, we set out to test these cells in a more complex neural organoid model. For that purpose, we generated Sox1^+^ neural spheres (Figs 5A and S7) as well as spheres formed by Brachyury^+^ MPCs (Figs 5B and S7) which were brought in co-culture. When a fusion of neural and mesodermal spheres was observed, these aggregates were isolated and cultured for up to 280 days in suspension culture on a cell culture rocking plate. Again, we tested the influence of hypoxic conditions on the formation of vascular structures, but found, in contrast to the tumor organoid, no major impact (Fig. S8). After 60 days, we observed a neuroepithelial tissue in direct contact with mesenchymal cells. The neuroepithelium is characterized by the expression of Pax6 (Fig. 5F–G) and Sox1 (Fig. 5H,I), while the mesenchyme remains negative for these markers. Within the mesenchymal tissue, vascular structures lined by CD31^+^ endothelial cells are found which clearly show lumen formation (Fig. 5F,G,J–K). Moreover, TUJ1^+^ neurons are observed at the interface between neuroepithelium and mesenchyme marking the basal side of the epithelium (Fig. 5H–K). At the border between neuroepithelium and mesenchyme, an extensive clustering of capillary-like structures is observed that closely resembles the formation of the perineural plexus, naturally found surrounding the neural tissue during embryogenesis^25^ (Fig. 5J,K). The capillaries within a 60 days old organoid (Fig. 5K) show remarkable similarity with those at the interface between mesenchyme and neuroectoderm in a developing chicken embryo at ED 5 (Fig. 5L).

Overall, we observe a strong vascularization of the mesodermal part of the organoids, showing a continuous and branching endothelial network including a hierarchy of larger and smaller vascular structures (Fig. 5M, Online Movie 3). The vessel structures show clear lumen formation (Fig. 5N). Electron microscopy demonstrates that the lumen is lined by cells with a typical endothelial cell morphology (Fig. 5O). In addition, periendothelial cells are found closely associated and sometimes even directly connected to the endothelium *via* finger-like processes, as naturally observed at contact sites of endothelial cells and smooth muscle cells (the so-called myoendothelial junctions)^26,27^ or pericytes^28,29^ (Figs 5O,P, S9A,B). Some periendothelial cells stain positive for pericyte markers NG2 and αSMA (Fig. S9C,D). Furthermore, the endothelial cells are polarized and the basal side is attached to a basal lamina (Fig. 5P). Neighbouring endothelial cells are tightly connected by characteristic junctional complexes (Fig. 5Q) and multiple caveolae are found at the luminal side, a typical hallmark of endothelial cells^30^ (Fig. 5R). At the luminal surface, microvesicles are observed that are released into the vessel lumen^31^ (Fig. 5S).

**Figure 6:**
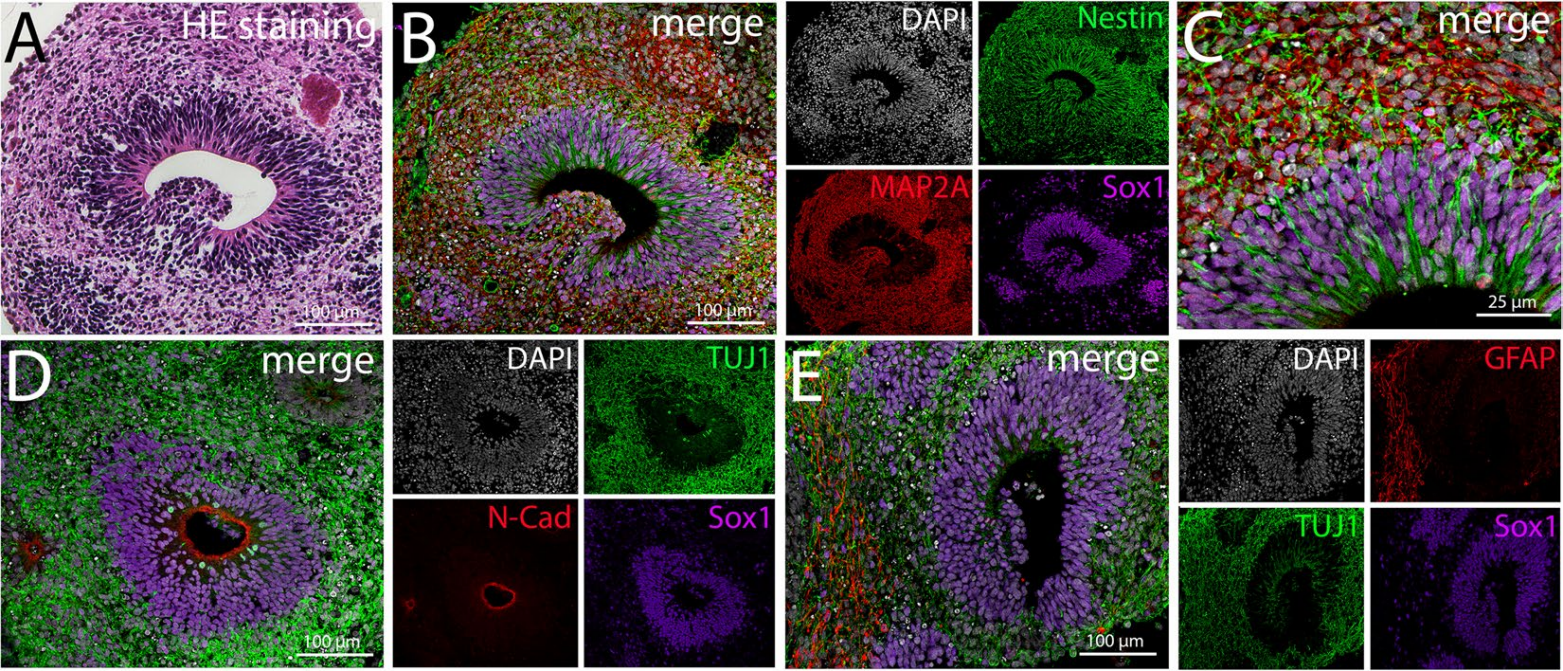
Characterization of the neural part of the organoid. Pictures show organoids at culture day 210. (**A**) HE-stained sections reveal ventricle-like cavities inside the neural tissue. (**B**–**C**) Neuroepithelial cells lining the ventricle-like structures stain positive for Sox1 and Nestin, while the cells surrounding the stem cell zone express the neuronal marker protein MAP2. (**C**) Shows a higher magnification of B. (**D**) The apical side of the neuroepithelium is marked by N-Cadherin^+^ cell-cell junctions. TUJ1^+^ cells can be found towards the basal side indicating neuronal differentiation. (**E**) Besides TUJ1^+^ neurons, GFAP^+^ radial glia cells or astrocytes can be found located towards the basal side of the epithelium.

Upon further differentiation, the neural part of the organoid self-organized and formed a well-structured neuroepithelium. The epithelium is located around fluid filled cavities (Fig. 6A). These ventricle-like structures are lined by Sox1^+^/Nestin^+^ stem cells (Fig. 6B,C) and show N-Cadherin^+^ cell-cell junctions marking the apical side of the neuroepithelium (Fig. 6D). Towards the basal side TUJ1^+^/MAP2a^+^ cells can be found indicating neuronal differentiation (Fig. 6B–D). Moreover, from day 180 onwards, GFAP^+^ cells are detected representing either radial glia cells or astrocytes (Fig. 6D).

**Figure 7:**
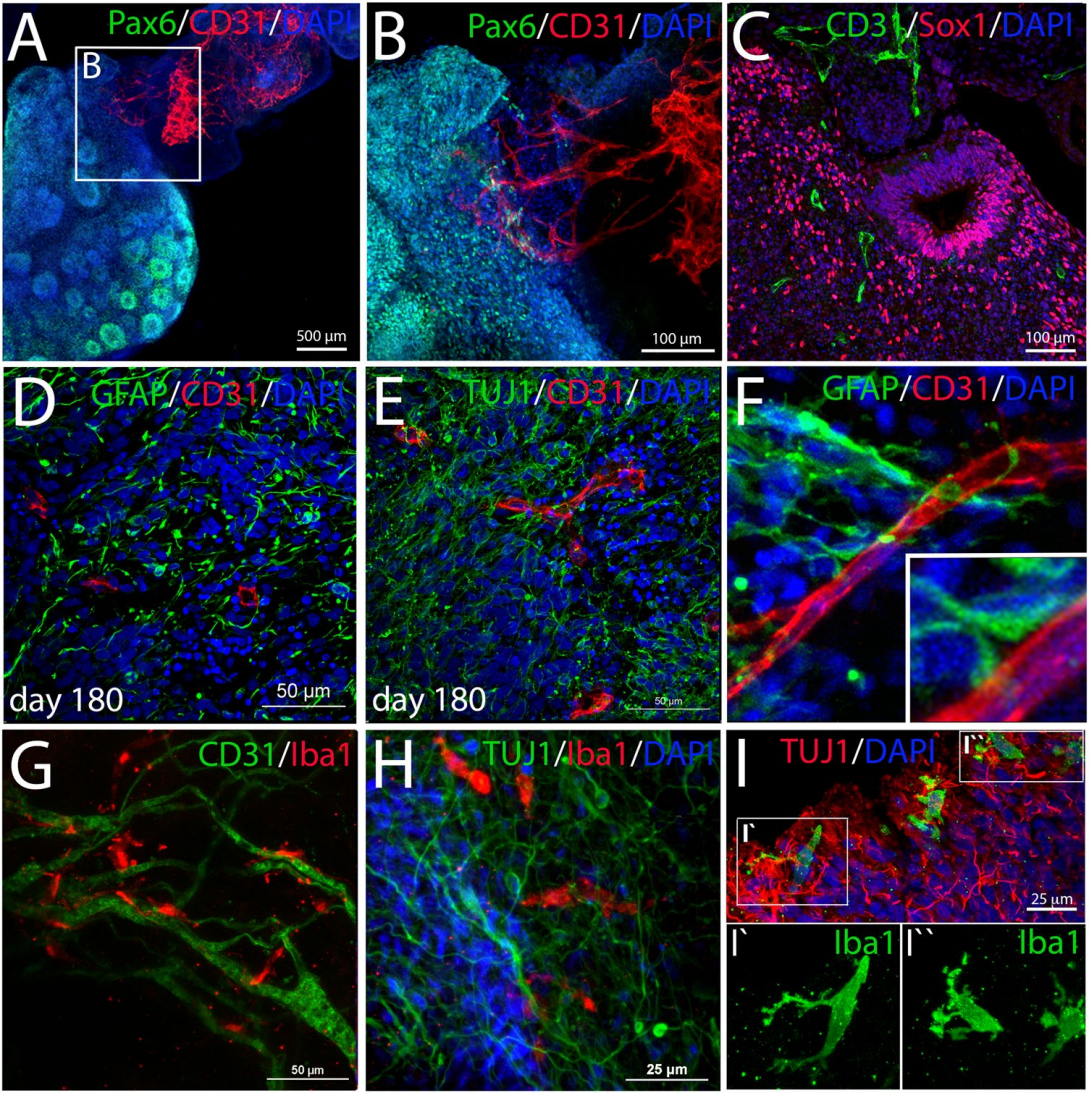
Vascularization of the neural part of the organoid and invasion of microglia-like cells. (**A**) At day 180 a CD31^+^ endothelial network infiltrating the Pax6^+^ neural part of the organoid was observed. (**B**) shows a higher magnification of A. (**C**) Infiltrating vessels were found within the neural tissue surrounding Sox1^+^ neuroepithelial structures. (**D**–**E**) CD31^+^ capillaries within the neural part of the organoid closely associated with GFAP^+^ astrocytes/radial glia and TUJ1^+^ neurons. (**F**) GFAP^+^ cells form close contacts with CD31^+^ endothelial cells (see inset). (**G**) Iba1^+^ macrophage-like cells were observed in close association with CD31^+^ capillaries within the mesodermal part of the organoid. (**H**) Iba1^+^ cells were also found within the neural part of the organoid being tightly associated with TUJ1^+^ neurons suggesting a microglia-like identity. (**I**) Microglia-like cells show a cellular morphology similar to microglial cells at the transition from an amoeboid to a ramified appearance. (**A**,**B**,**F**,**G**,**H**) Show maximum intensity projections of whole-mount stained cleared organoids. (**C**,**D**,**E**,**I)** show stained paraffin sections.

Finally, we were interested, if angiogenic invasion occurs from the perineural plexus into the nervous tissue. After 180 days of culture, we observed CD31^+^ structures invading the neural tissue at the interfaces between neural and mesodermal parts of the aggregate (Fig. 7A,B). CD31^+^ sprouts were detected in the neural tissue close to Sox1^+^ neuroepithelial structures (Fig. 7C). Immunofluorescence analyses confirmed the presence of CD31^+^ capillaries within the GFAP^+^/TUJ1^+^ neural part of the organoids (Fig. 7D,E). CD31^+^ endothelial cells are in close association with GFAP^+^ cellular processes showing direct contact sites (Figs 7F, S10A). Such GFAP^+^ cells could be either astrocytes or radial glia cells. Astrocytes contact blood vessels via foot processes, contributing to blood brain barrier formation, while radial glia cells extend long basal processes with specialized end feet contacting blood vessels^32,33^. Both cell types express GFAP in humans.

Interestingly, Iba1^+^ cells with amoeboid morphology are frequently observed lining the perivascular space within the mesodermal part of the organoids reminiscent of perivascular macrophages^34^ (Fig. 7G). Iba1^+^ cells are also found to invade the neural part of the aggregates as observed during microglial invasion in embryonic development (Fig. 7H). The Iba1^+^ cells display a similar cellular morphology as early microglia, changing from an amoeboid to a ramified phenotype^35^ (Fig. 7I). Moreover, we observed that extensive vascular networks form at sites of retinal pigmented epithelium (RPE) which is frequently found within the neural part of the organoids, probably attracted by VEGF produced by the cells of the RPE (Fig. S10B)^36^.

Our initial experiments for the production of vascularized neural organoids led to variable results, especially regarding organoid size and the extent of vascularization. This is a frequently observed issue in organoid cultures and the problem of poor reproducibility increases with the complexity of the structure and the duration of culture time^37–39^. For that reason, we refined the protocol as follows to increase reproducibility (Fig. S11). We first produced neural and mesodermal aggregates of defined size. This was achieved by pipetting 4 * 10^3^ cells into each agarose-coated non-adhesive well of a 96-well plate. At day 5 of neural and day 4 of mesodermal induction, a neural aggregate is transferred into a well already containing a mesodermal aggregate. The aggregates are then co-cultured. After 24–48 h, aggregates attach to each other and form dumbbell-like structures (Fig. S11B). These are embedded into basement membrane extract (BME) and transferred to a 6 cm dish (Fig. S11C). The aggregates are further cultured on a rocking plate in the humidified incubator (Fig. S11D). Using this improved protocol, we produce aggregates of comparable size and cellular composition (Fig. S11D). Although, we still observe a certain degree of variability between different experiments, each of the generated aggregates consists of a vascularized mesodermal and a neural part (Fig. S11DE–G). We repeated the protocol with a second iPS cell line (Sendai NHDF iPSCs) which led to a similar result (Fig. S11H).

## Discussion

In order to bring organoids closer to the original tissue architecture and to create a complex model with all important niches relevant for developmental and pathological processes, the incorporation of stromal components such as connective tissue and tissue resident immune cells as well as functional vasculature is essentially required. This might also have an impact on disease modelling, e.g. regarding neurodegenerative disorders such as Parkinson’s or Alzheimer’s disease, in which the importance of healthy brain vasculature is increasingly recognized^40^.

Recent reports addressed the issue of organoid vascularization making use of different strategies. Human neural organoids were transplanted into the mouse brain which resulted in vascularization by the host and immigration of microglia into the graft^12^. Although, this is a very attractive model, neural and stromal components are not derived from the same species and require transplantation into a living animal. Another report described vascularization of neural organoids making use of iPSC-derived neural organoids and autologous endothelial cells^11^. But blood vessels are more complex than a simple endothelial tube. They consist of endothelial cells, smooth muscle cells or pericytes as well as adventitial connective tissue which cannot derive from endothelial cells alone, but are delivered from a plastic mesodermal progenitor, the angioblast, as well as the surrounding mesenchyme.

Here, we show the feasibility to generate human vascularized organoids by mixing with MPCs or co-culturing with mesodermal aggregates derived thereof. First, we generated tumor organoids which are highly uniform in size and distribution of the endothelial network, can be produced in great quantities and generated within only 10–14 days, making them an interesting model for drug screening. Moreover, we observed a high plasticity in the endothelial network which expanded while the tumor aggregates grew, showed a hierarchic organization and was responsive to anti-angiogenic compounds and pro-angiogenic conditions such as hypoxia.

In a second approach, we generated complex vascularized neural organoids that recapitulate several aspects of development e.g. formation of a perineural plexus and invasion of endothelial sprouts and microglia. While simpler *in vitro* generated tissues, such as vascularized tumor spheroids or cardiac patches, can be also generated by mixing tumor cells or cardiomyocytes with endothelial cells and mesenchymal stem cells^41–43^, complex tissues are generated by sequentially recapitulating developmental steps similar to the situation observed in the embryo. For that reason, we propose that the co-culture of neuroepithelium and embryonic mesenchyme creates an important environment similar to the *in vivo* situation in which a defined interface between mesenchyme and neuroepithelium can be found. The mesenchyme finally forms the meninges that start covering the neuroepithelium. The mesenchyme essentially contributes to the pial basement membrane as anchor point for the projections of radial glia cells. Moreover, it secretes factors that influence neural progenitor migration and proliferation^9^. Similar developmental mechanisms were recently described in liver organoids consisting of hepatocytes, mesenchymal cells and endothelial cells^44,45^. The authors demonstrate that multilineage communication between stromal, endothelial and hepatic cell types regulates organoid development and maturation.

In addition, the mesenchymal/epithelial interaction triggers the formation of the perineural vascular plexus, the initial step in neural vascularization^25^. While the pial basement membrane has been mimicked in existing organoid models by encapsulating them in basement membrane extracts^5^, the other aspects have not been addressed yet. Finally, the mesenchyme does not only produce vasculature but also hematopoietic cells. This is important, especially with regard to neural tissue, as blood islands during early development are the source of the microglial cell pool. Although, microglia naturally emerge from yolk sac blood islands^46^, our data suggests that blood islands developing within the MPC-derived mesenchyme might, in the context of our organoid model, deliver microglia-like cells in a similar way^14^.

**Figure 8:**
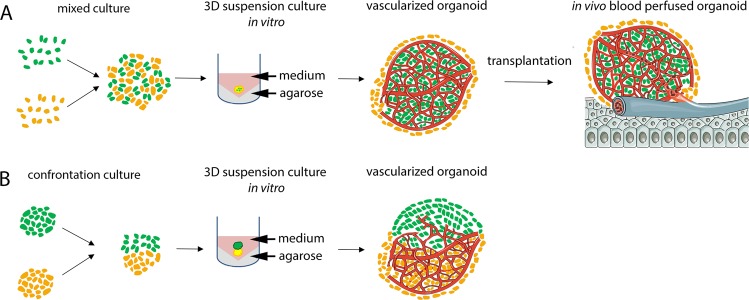
Schematic representation of the workflow. Generation of vascularized organoids is achieved by (**A**) direct mixing or (**B**) co-culturing of MPC with tissue specific (progenitor) cell types, e.g. tumor cells or early neuroepithelial stem cells. The organoids form and mature in suspension culture. Prevascularized organoids can be transplanted on the chicken chorion allantois membrane, where the already established human vessels connect to the host circulation (**A**). Parts of this schematic were adapted from Servier Medical Art licensed under a Creative Commons Attribution 3.0 Unported License (https://creativecommons.org/licenses/by/3.0). Excerpts of the original images were used and colour was edited.

Our data show the generation of *in vitro* pre-vascularized organoids by self-organization of two co-cultured cell types. After transplantation on the chicken CAM, the *in vitro* generated vessels connect to the host circulation within few days and preserve their vessel structure. Thus, besides representing an improved *in vitro* platform for the modelling of development and disease, the presented technique could also serve as a basis for the generation of pre-vascularized complex tissue pieces that can be used for long term *in vivo* disease modelling or even replacement therapies (Fig. 8). Exploring strategies for the generation of more uniform vascularized neural organoids and their long-term connection to a circulatory system are important challenges to be solved in future experiments. Perfusion of the vascular network is required to promote further maturation of the pre-formed organoid vessels. We could already detect many aspects of vessel maturation within the organoid model, such as a hierarchic and branching network, a basement membrane, endothelial cell-cell junctions, luminal caveolae, microvesicle release, recruitment of periendothelial cells and responsiveness to pro- and antiangiogenic stimuli. However, it is well known that shear stress, caused by pulsatile blood flow, is sensed by the cells of the endothelium and triggers multiple biological responses in endothelial cells as well as surrounding smooth muscle cells and pericytes, inducing the final maturation of the vessel wall^47^. Stable long-time perfusion could be achieved by transplantation into a suitable host organism, e.g. a mouse, or ideally by connecting vascularized organoids to a perfused artificial vascular network^43^. Such *in vitro* models would allow for an easy manipulation of the system and can be live monitored. Moreover, high throughput platforms could be developed. This would provide a robust model system for studying angiogenesis and neural development as well as diseases affecting the nervous system and drug testing applications.

## Methods

### Cell Culture

Two independent lines of human foreskin derived male iPSCs were used (STEMCCA NHDF iPSCs and Sendai NHDF iPSCs). iPS cells were generated from commercially available normal human dermal fibroblasts (juvenile NHDF, C-12300, Promocell, Heidelberg, Germany) by reprogramming, using either the hSTEMCCA‐lentiviral construct^48^ or a Sendai virus reprogramming kit (Cyto Tune 2.0 Sendai reprogramming vectors, Ref#A16517; Lot#A16517, Invitrogen, Carlsbad, CA). The STEMCCA NHDF iPS cell line was kindly provided by Dr. Frank Edenhofer, University of Innsbruck and has been already published before^49^. Basic characterization of the Sendai NHDF iPS cell line is shown in Fig. S2. iPS cells were cultured on Matrigel coated culture dishes in StemMACS iPS-Brew medium (Myltenyi Biotec, Bergisch Gladbach, Germany). For MPC induction 5 × 10^4^ iPSCs per cm^2^ were seeded on Matrigel coated dishes and cultured for 1 day in StemMACS iPS-Brew medium with 10 µM ROCK-inhibitor (Y-27632). Subsequently, medium was changed to mesodermal induction medium (Advanced DMEM/F12, 0.2 mM L-glutamate, 60 µg/µl Vit.C, 10 µM CHIR 99021, 25 ng/ml BMP4) and cells were cultured for 72 h. Medium was changed every day. To induce endothelial (EC) or smooth muscle cells (SMC), differentiation medium was changed to EC induction medium (StemSpan SFEM, 200 ng/ml VEGF, 0.2 mM forskolin) or SMC induction medium (Advanced DMEM/F12, 0.2 mM L-Glutamate, 60 µg/ml Vit.C, 10 ng/ml PDGF-BB, 2 ng/ml Activin A). After 2 days endothelial cells were detected. Smooth muscle cells were detectable at day 4.

A method for vascularized tumor organoids was developed based on a liquid overlay protocol^50,51^: A MPC single cell suspension was prepared. 2 × 10^3^ MPCs were mixed in 1:1 ratio with GFP-labelled MDA-MB-435s cells (Fig. S1 A). The cell suspension was transferred into an agarose coated well of a 96-well plate. To prepare agarose coated wells, 1% agarose is boiled in water and 50 µl liquid gel are pipetted into each well of the 96-well plate. Aggregates were cultured for 24 h in a humidified incubator with 20% O_2_ (5% CO_2_) in tumor organoid medium (Advanced DMEM/F12, 0.2 mM L-Glutamate, 60 µg/µl Vit.C, 10% FCS, 100 ng/ml VEGF, 1% Penicillin/Streptomycin, 10 µm ROCK-inhibitor (Y-27632)). Afterwards, cells were placed for 3 days in an incubator with 2% O_2_ (5% CO_2_). Aggregates were grown for up to 10 days.

For the generation of vascularized neural organoids, neural and mesodermal aggregates were prepared separately and afterwards brought in co-culture. First, 7 × 10^5^ iPSCs were seeded per well of a non-adhesive 6-well plate (Nuclon Sphera, Thermo Scientific) and grown overnight in 3D-suspension culture. For neural induction, aggregates were grown for 2 days in neural induction medium 1 (DMEM/F12 / Neurobasal (50:50), 1x N2 supplement, 1x B27 (w/o Vitamin A), 1% L-Glutamate, 10 µM SB431542, 1 µM Dorsomorphin, 3 µM CHIR 99210, 0.5 µM Purmorphamine) and afterwards for 3 days in neural induction medium 2 (DMEM/F12 / Neurobasal (50:50), 1x N2 supplement, 1x B27 (w/o Vitamin A), 1% L-Glutamate, 3 µM CHIR 99210, 0.5 µM Purmorphamine). From day 5 on aggregates were grown in neural differentiation medium (DMEM/F12 / Neurobasal (50:50), 1x N2 supplement, 1x B27 (w/o Vitamin A), 1% L-Glutamate, 60 µg/µl Vit.C). For mesodermal induction, iPSC aggregates were cultured for 3 days in mesodermal induction medium.

At day 4 of mesodermal induction and day 5 of neural induction, aggregates were co-cultured in neural differentiation medium for up to 280 days on a rocking plate in a humidified incubator (20% O_2_, 5% CO_2_).

### Immunofluorescence analyses

For immunofluorescence analyses, vascularized organoids were fixed in 4% PFA solution for 30 min, washed in PBS and embedded into an agarose gel (1%). Afterward, 5 µm paraffin sections were prepared. Sections were deparaffinized, rehydrated and stained with eosin and hematoxylin. For immunofluorescence analyses, antigens were unmasked using Sodium Citrate buffer (10 mM, pH6). Primary antibodies to TUJ1 (Biozol, 801202), GFAP (DAKO, Z0334), CD31 (DAKO, M0823), Iba1 (WAKO, 019-19741), Sox1 (R&D Systems, AF3369), Pax6 (Biolegend, 901301), Brachyury (T) (R&D Systems, AF2085), MAP2 (Abcam, AB32454), N-Cadherin (Sigma-Aldrich, C3865) and NG2 (Merck-Millipore, AB5320) were used. Sections were incubated with primary antibodies overnight at 4 °C. Secondary Cy2‐, Cy3- or Cy5-labelled antibodies were used to visualize primary antibodies. Sections were incubated with secondary antibodies for 2 h at room temperature. All antibodies were diluted in blocking solution.

### Tissue clearing analyses

Organoids were harvested, washed in PBS and fixed in 4% PFA in PBS for 1 h at RT. Afterwards, organoids were washed 3x for 30 min in PBS. Subsequently, organoids were incubated in an ascending MeOH series (30 min 50% MeOH in PBS, 30 min 80% MeOH in PBS, 2 × 30 min 100% MeOH). Samples were washed 2 × 30 min in 20% DMSO in MeOH. Next, samples were incubated in a descending MeOH series (30 min 80% MeOH in PBS, 30 min 50% MeOH in PBS, 30 min PBS) and incubated 2 × 30 min in 0.2% Triton-X-100 in PBS. Then, samples were incubated in penetration buffer (PBS, 0.2% Triton X-100, 0.3 M Glycine, 20% DMSO) at 37 °C over night. Subsequently samples were incubated in blocking solution (PBS, 0.2% Triton X-100, 0.3 M Glycine, 6% BSA, 10% DMSO) for 16 h at 37 °C. Next, samples were washed 2x for 1 h in washing buffer (0.2% Tween-20 in PBS) and afterwards incubated with primary antibodies in antibody buffer (PBS, 20% Tween-20, 4% BSA, 5% DMSO) for 24 h at 37 °C. Following primary antibodies were used: GFAP (DAKO, Z0334), CD31 (DAKO, M0823), Iba1 (WAKO 019-19741) and Pax6 (Biolegend, 901301). Next samples were washed twice for 30 min in washing buffer. Incubation with secondary antibodies was performed over night at 37 °C in antibody buffer. Samples were washed 3 times for 30 min in washing buffer, 3 times for 30 min in 50% MeOH in PBS, 3 times for 30 min in 80% MeOH in PBS and 3 times for 30 min in 100% MeOH. Finally, samples were incubated in ethyl cinnamate for 24 h at 37 °C.

### Immunoblot analyses

For immunoblot analyses, cells were lysed with RIPA buffer (150 mM NaCl, 1% Triton X, 0.5% sodium deoxycholate, 0.1% SDS, 50 mM Tris (pH 8.0)). Proteins were separated by SDS‐polyacrylamide gel electrophoresis and transferred on nitrocellulose membrane. The membrane was blocked using 5% milk powder in wash buffer (8.5 mM Tris‐HCl, 1.7 mM Tris‐Base, 50 mM NaCl, 0.1% Tween 20 in a. bidest) for 1 h and incubated with primary antibodies against Brachyury (T) (R&D Systems, AF2085) and β-Actin (Sigma Aldrich, A1978) for 2 h at RT. Secondary horseradish peroxidase‐conjugated antibodies were used to detect primary antibodies. Antibodies were diluted in blocking solution. To visualize bound antibodies, Luminol solution was used.

### Flow cytometric analyses

Flow cytometric analyses were performed using a *FACSCanto II* and obtained data analyzed using FACSDiva software (version 6.1.3) (BD Biosciences, USA). To discriminate viable from apoptotic cells, 1 µl of fixable viability stain 450 (BD Biosciences, CA, USA) was added and incubated for 15 min. After that, cells were fixed with 4% PFA in PBS, washed twice with PBS and finally incubated for 1 h at RT in blocking buffer (5% BSA in PBS). Cells were permeabilized using blocking buffer containing 0.1% Triton X-100. Afterwards, cells were incubated with primary antibodies targeted against Brachyury (T) for 1 h at RT. Antibodies were diluted in blocking buffer. After 3 washes with PBS, cells were incubated with corresponding anti-IgG secondary antibodies in PBS at RT for 45 min. Unstained cells and cells treated with secondary antibodies alone served as negative controls.

### Transplantation on chicken CAM

10–20 tumor organoids were transferred in 10 µl Matrigel and placed on a 1.5 × 1.5 cm nylon mesh (150 µm grid-size, 50% open surface, 62 µm string diameter, 35 g/m^2^, PAS2, Hartenstein, Germany). After gelling, the mesh was placed upside down on the CAM of a developing chicken embryo at day 7 of development. After 3 days the mesh was cut out from the CAM, washed in PBS and fixed for 1 h in 4% PFA. Subsequently, 5 µm paraffin sections were prepared and processed for immunofluorescence analyses as described above. All procedures complied with the regulations covering animal experimentation within the EU (European Communities Council DIRECTIVE 2010/63/EU). They were conducted in accordance with the animal care and use guidelines of the University of Würzburg. *In ovo* experiments do not require any special additional allowance as long as the embryos are sacrificed before hatching as is done in this study.

### Transmission electron microscopy

Organoids were fixed in 2.5% glutaraldehyde, 4% PFA in 0,1 M cacodylate buffer (50 mM cacodylate, 50 mM KCl, 2. 5 mM MgCl2, pH 7.2) on ice for 2 h and washed 4 times with 0,1 M cacodylate buffer, 5 min each. Organoids were subsequently fixed for 60 min with 1% osmium tetroxide in 0,1 M cacodylate buffer and washed for 2 times with 0,1 M cacodylate buffer, 10 min each. After washing with aqua bidest, organoids were dehydrated in an ascending EtOH series using solutions of 30%, 50%, 70% EtOH, 10 min each. Organoids were contrasted with 2% uranyl acetate in 70% EtOH for 60 min and subsequently dehydrated using an EtOH array of 70%, 80%, 90%, 96% and two times 100% for 10 min each. Subsequently, organoids were incubated in propylene oxide (PO) two times for 30 min before incubation in a mixture of PO and Epon812 (1:1) overnight. The following day samples were incubated in pure Epon for 2 h and embedded by polymerizing Epon at 60 °C for 48 h.

Specimen were cut with an ultracut, collected on nickel grids and post-stained with 2.5% uranyl acetate and 0,2% lead citrate and finally analyzed with a LEO AB 912 transmission electron microscope (Carl Zeiss Microscopy GmbH, Germany).

## Electronic supplementary material

Supplementary video 1

Supplementary video 2

Supplementary video 3

Supplementary Information
