# Supplementary material for: Generation of complex human organoid models including vascular networks by incorporation of mesodermal progenitor cells
Source: Sci Rep. 2019 Oct 30;9:15663. doi: 10.1038/s41598-019-52204-7 (PMC6821804; doi:10.1038/s41598-019-52204-7)
Supplement: Supplementary file 4 — Supplementary Information [file 41598_2019_52204_MOESM4_ESM.pdf]

Supplemental Data

Generation of complex human organoid models including vascular networks by incorporation of mesodermal progenitor cells

Philipp Wörsdörfer[$], Nahide Dalda*, Anna Kern*, Sarah Krüger, Nicole Wagner, Chee Keong Kwok, Erik Henke, Süleyman Ergün

Institute of Anatomy and Cell Biology, Koellikerstraße 6, University of Würzburg, 97070 Würzburg, Germany

* authors contributed equally to this work
[$] correspondence to:

Dr. Philipp Wörsdörfer
Institute of Anatomy and Cell Biology
University of Würzburg,
97070 Würzburg
Germany
Mail: *philipp.woersdoerfer@uni-wuerzburg.de*
Phone: +49 931 31-80884

Figure S1

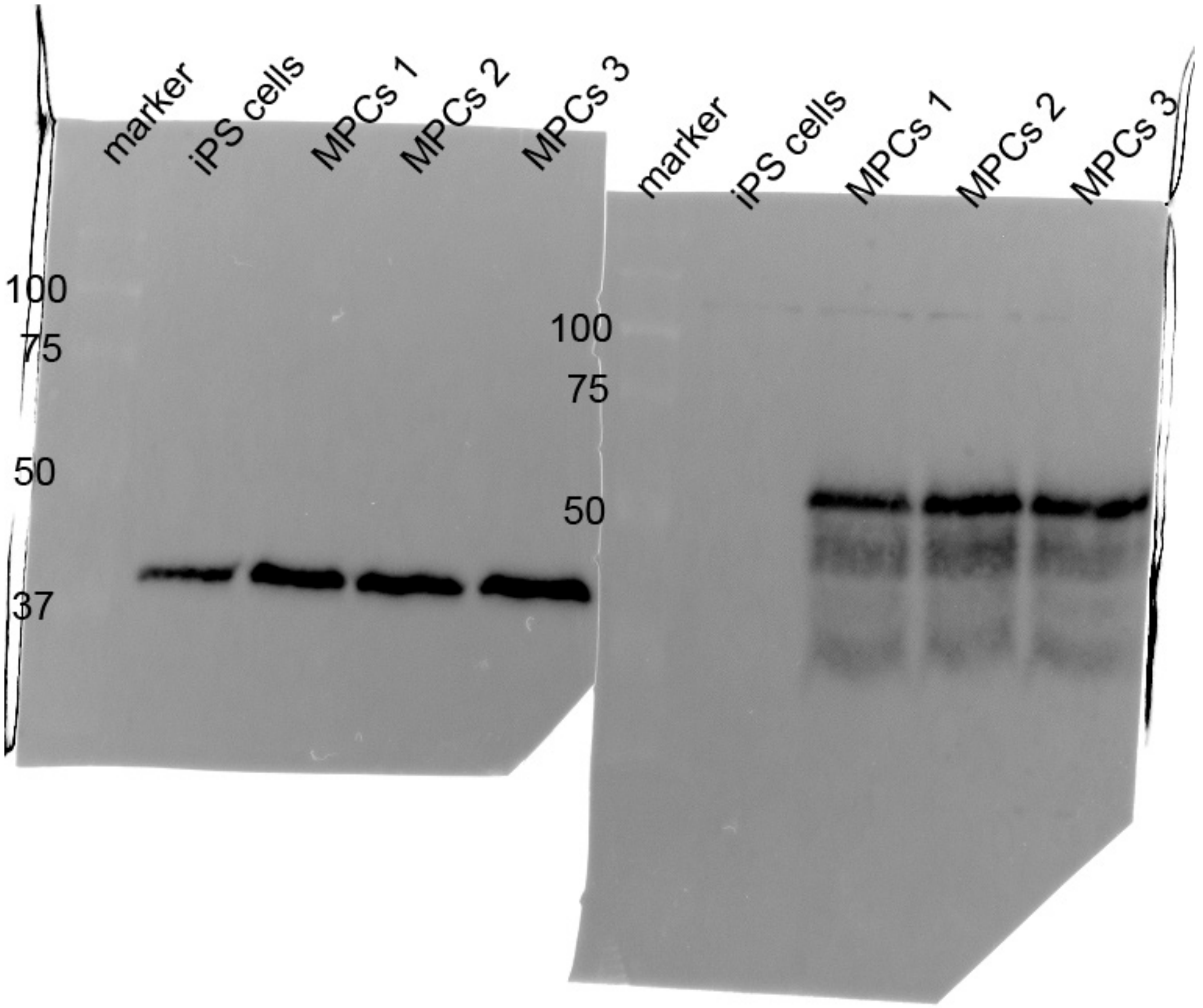

Figure S1: Full length blots for the detection of β-Actin and Brachyury (T).
Bry(T) expression was demonstrated performing immunoblot analyses (blot on the right side). As loading control β-Actin is detected (blot on the left side). Three independent inductions of MPCs from iPS cells (MPCs 1 - MPCs 3) are depicted.

Figure S2

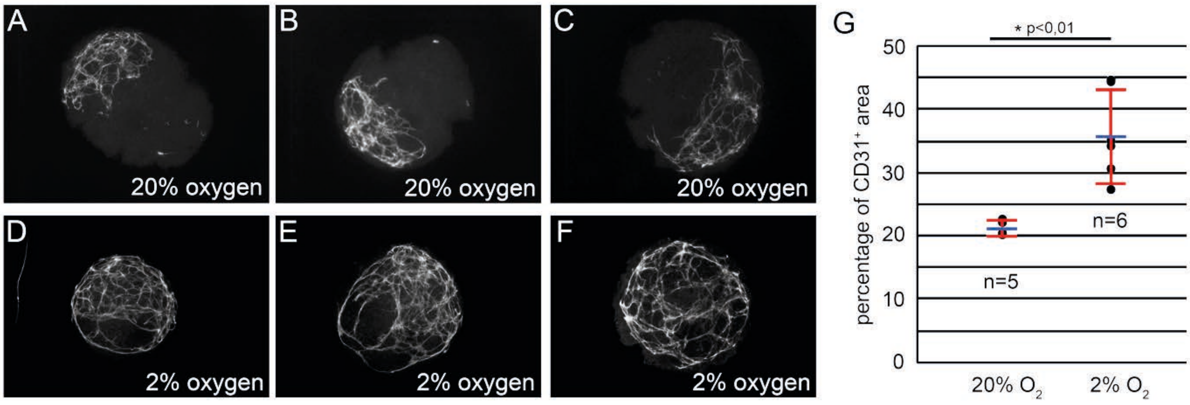

Figure S2: Effect of hypoxia on endothelial network formation in tumor organoids.
(A-F) Representative images to illustrate the effect of hypoxia (2% O_2) on endothelial network formation in tumor organoids. CD31 is detected using a specific antibody. Normoxic conditions (20% O_2) are depicted in A-C, hypoxic conditions in D-F (2% O_2). (G) Quantification of the organoid`s surface area covered by $CD31^+$ cells.

Figure S3

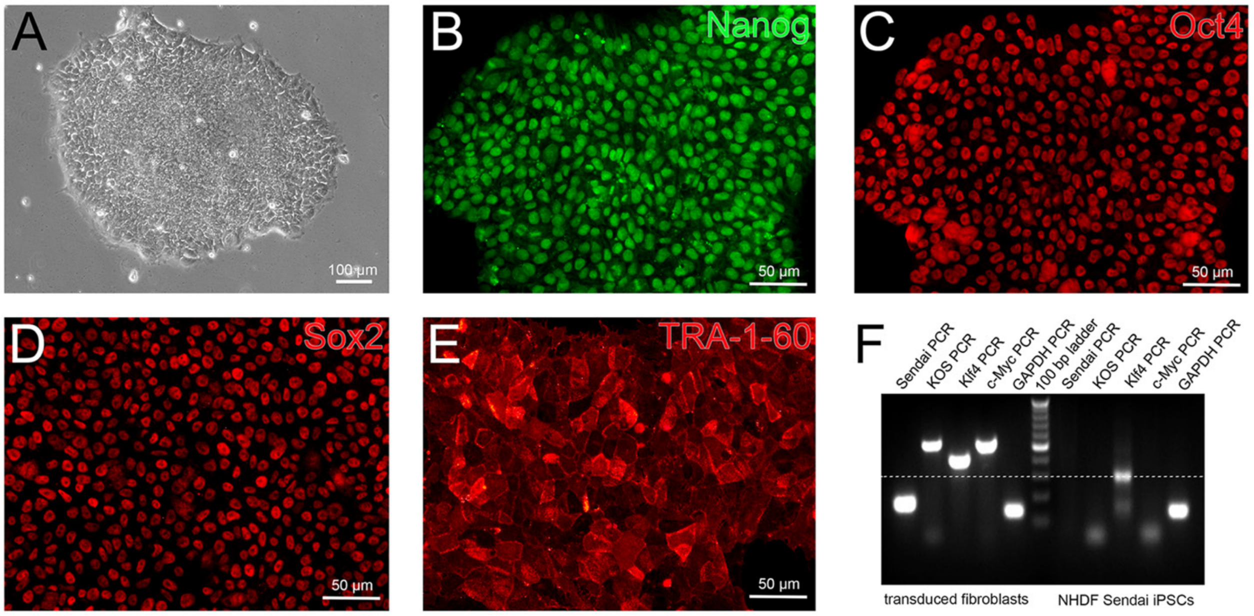

Figure S3: Characterization of Sendai NHDF iPS cells.
(A-F) To demonstrate that the protocol for the generation of vascularized human tumor organoids is also applicable for other iPS cell lines, we generated a new iPS cell line from NHDF fibroblasts using non-integrative Sendai virus-based vectors. (A) Colony morphology of Sendai NHDF iPSCs. (B-E) Detection of pluripotency markers Nanog, Oct4, Sox2 and TRA-1-60 in Sendai NHDF iPSCs. (F) Fully reprogrammed iPSCs do no longer contain Sendai viral particles as demonstrated by PCR analyses (right side). In contrast, virus transduced fibroblasts (left side) show clear bands in the PCR detecting Sendai viral particles (Sendai PCR) as well as the 3 individual viral particles used for reprogramming (KOS, Klf4, c-Myc). GAPDH was used as loading control. The unspecific band in the Klf4 PCR was also detected in untransduced control samples.

Figure S4

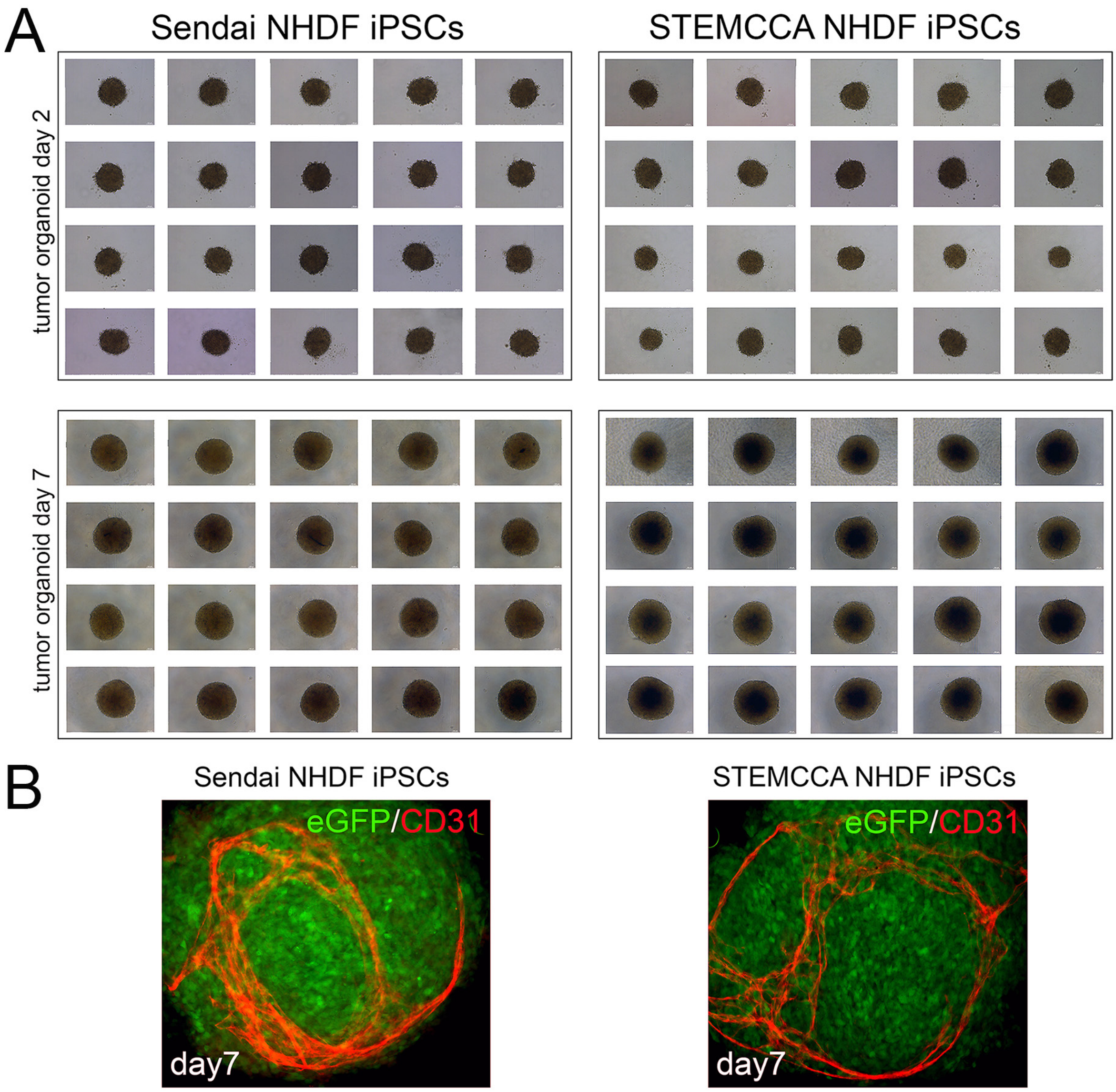

Figure S4: Reproducibility of the tumor organoid protocol.
To demonstrate that the protocol for the generation of vascularized human tumor organoids is applicable for different independently generated iPS cell lines, we generated tumor organoids from STEMCCA NHDF iPSCs and Sendai NHDF iPSCs. (A) Pictures of 20 randomly picked organoids at day 2 and day 7 are depicted. Organoids look undistinguishable regarding size and morphology between the different experiments. (B) $CD31^+$ vascular networks can be detected in tumor organoids derived from Sendai NHDF iPSCs as well as from STEMCCA NHDF iPSCs.

Figure S5

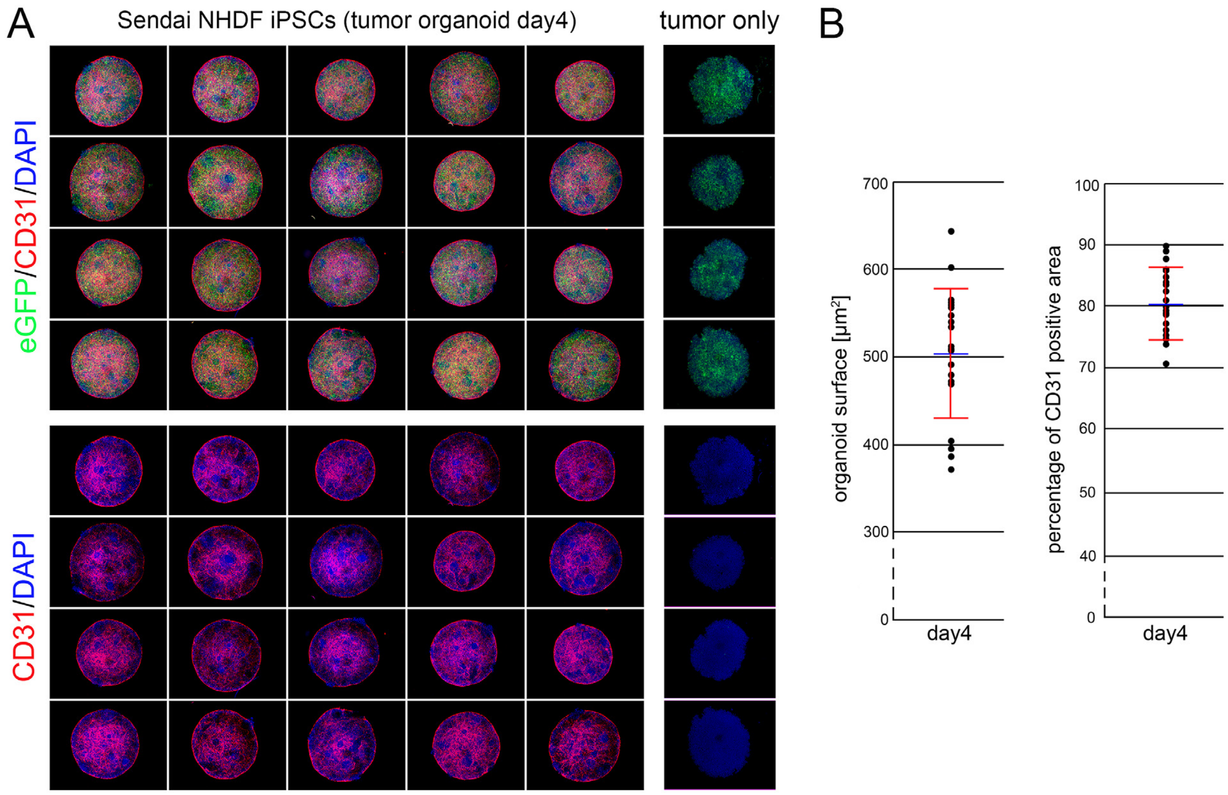

Figure S5: Variations in tumor organoids from the same experiment.
To investigate variations between vascularized tumor organoids from the same experiment, we randomly picked 20 organoids at day 4, performed CD31 immunofluorescence analyses and measured the organoid surface as well as the area covered by $CD31^+$ cells. (A) Immunofluorescence analyses of 20 organoids are depicted. Control spheroids consisting of tumor cells only do not show any $CD31^+$ cells. (B) The total organoid surface was quantified as well as the percentage of $CD31^+$ area. We measured a mean surface area of ca. 500 μm^2 (+/- 71.5 μm^2) and a percentage of $CD31^+$ area of 80% (+/-5.3%). Analyses were performed at whole mount stained organoids.

Figure S6

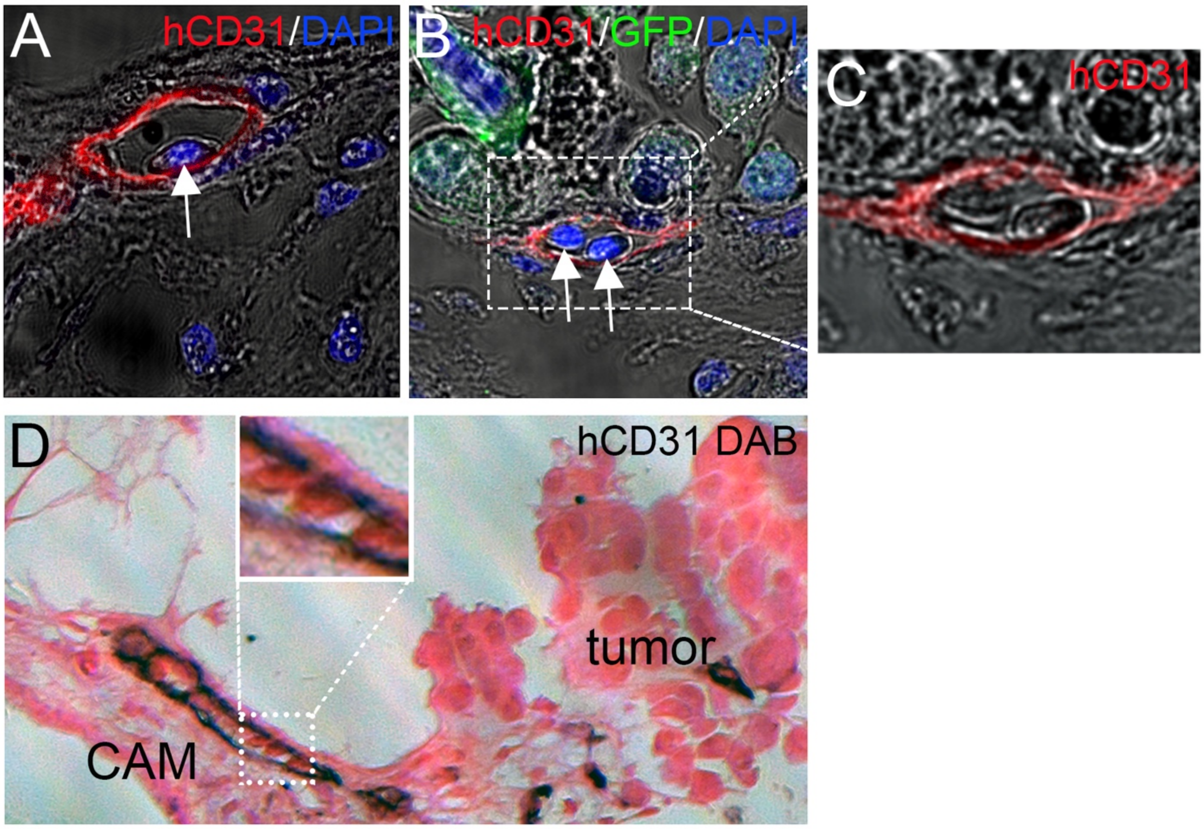

Figure S6: Chicken blood cells within human organoid vessels.

Tumor organoids were transplanted on the chicken chorioallantoic membrane and a connection of $CD31^+$ human vessels to $CD31^-$ chicken vessels was observed. (A-C) The pictures demonstrate chicken blood cells (white arrows) in the lumen of human vessels. C shows a higher magnification of B. (D) An immunohistochemical staining for the detection of CD31 (black) is shown. The picture shows a longitudinally sliced human blood vessel with chicken blood cells in the luminal space (see inset).

Figure S7

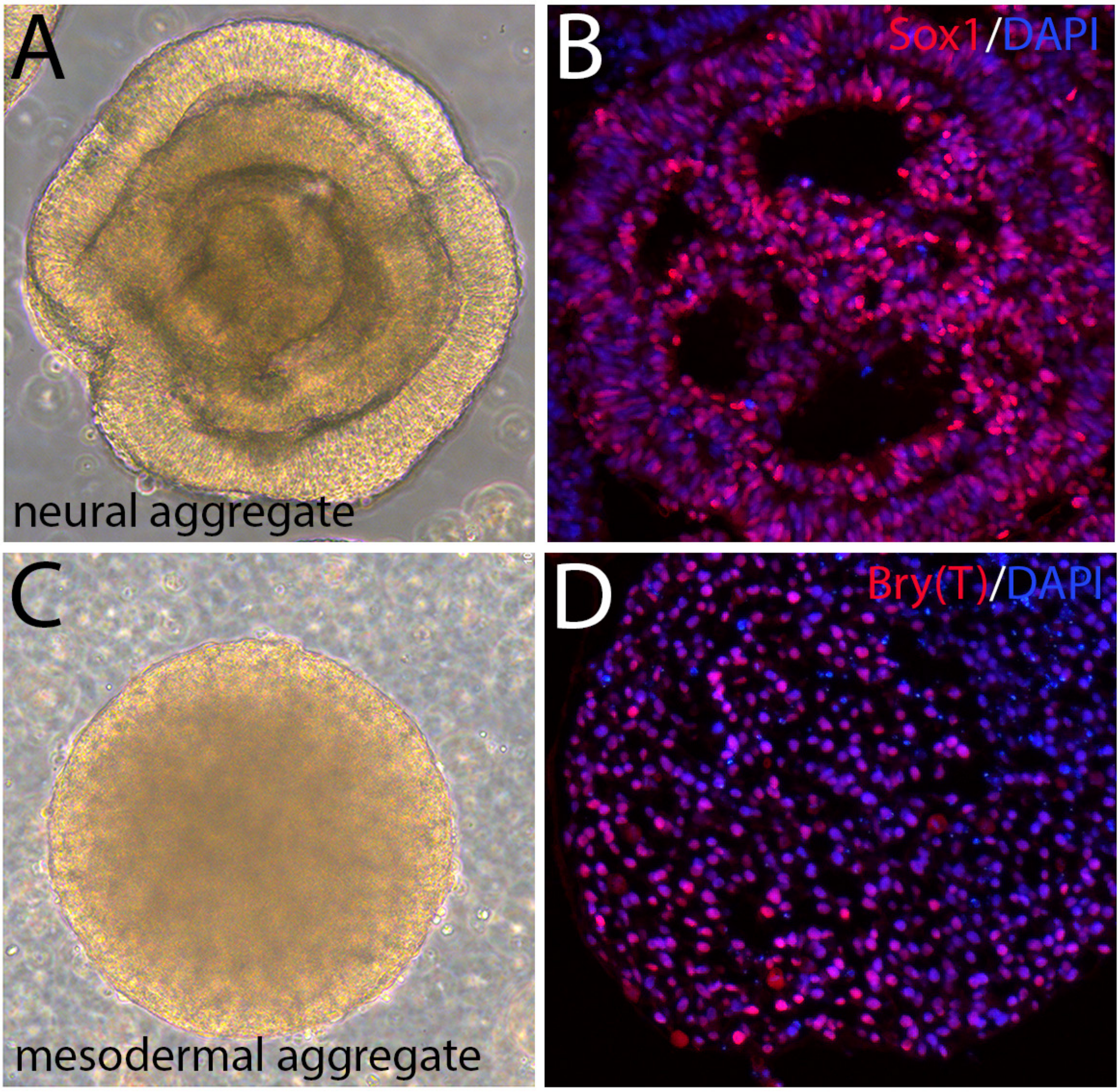

Figure S7: Confirmation of the neural and mesodermal identity of spheroids used for aggregation.
(A-B) Neuroepithelial spheres used for chimeric aggregate formation express the neuroepithelial transcription factor Sox1 and organize into a typical columnar epithelium. (C-D) Cells within the mesodermal aggregates show expression of the transcription factor Brachyury (Bry(T)).

Figure S8

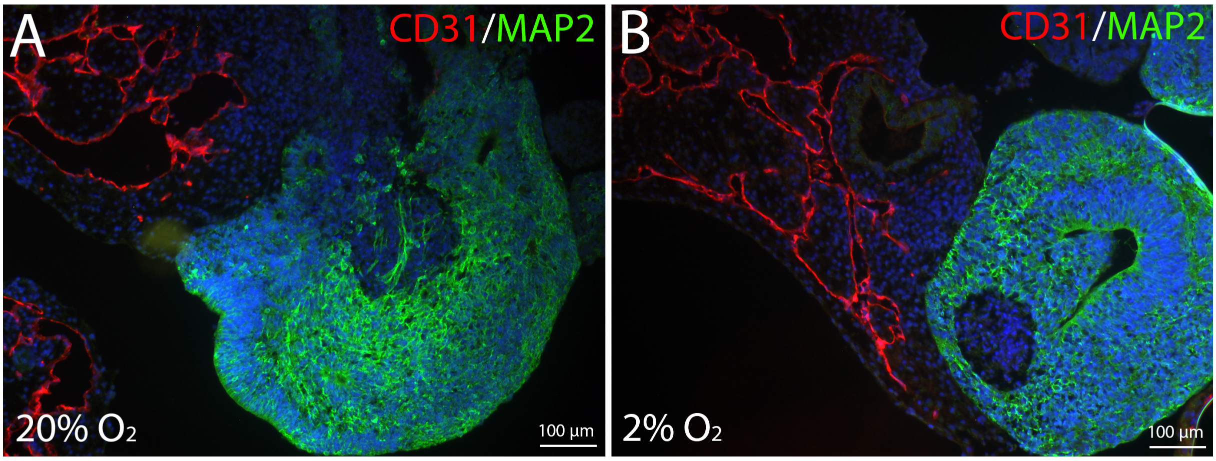

Figure S8: The effect of hypoxia on the formation of vascular structures in neural organoids.

The network of blood vessel-like CD31^{+} structures in chimeric aggregates grown during the initial 3 days of confrontation culture under normoxic conditions (A) was comparable to those aggregates grown in a hypoxic environment (B). Pictures were taken after 23 days in confrontation culture.

Figure S9

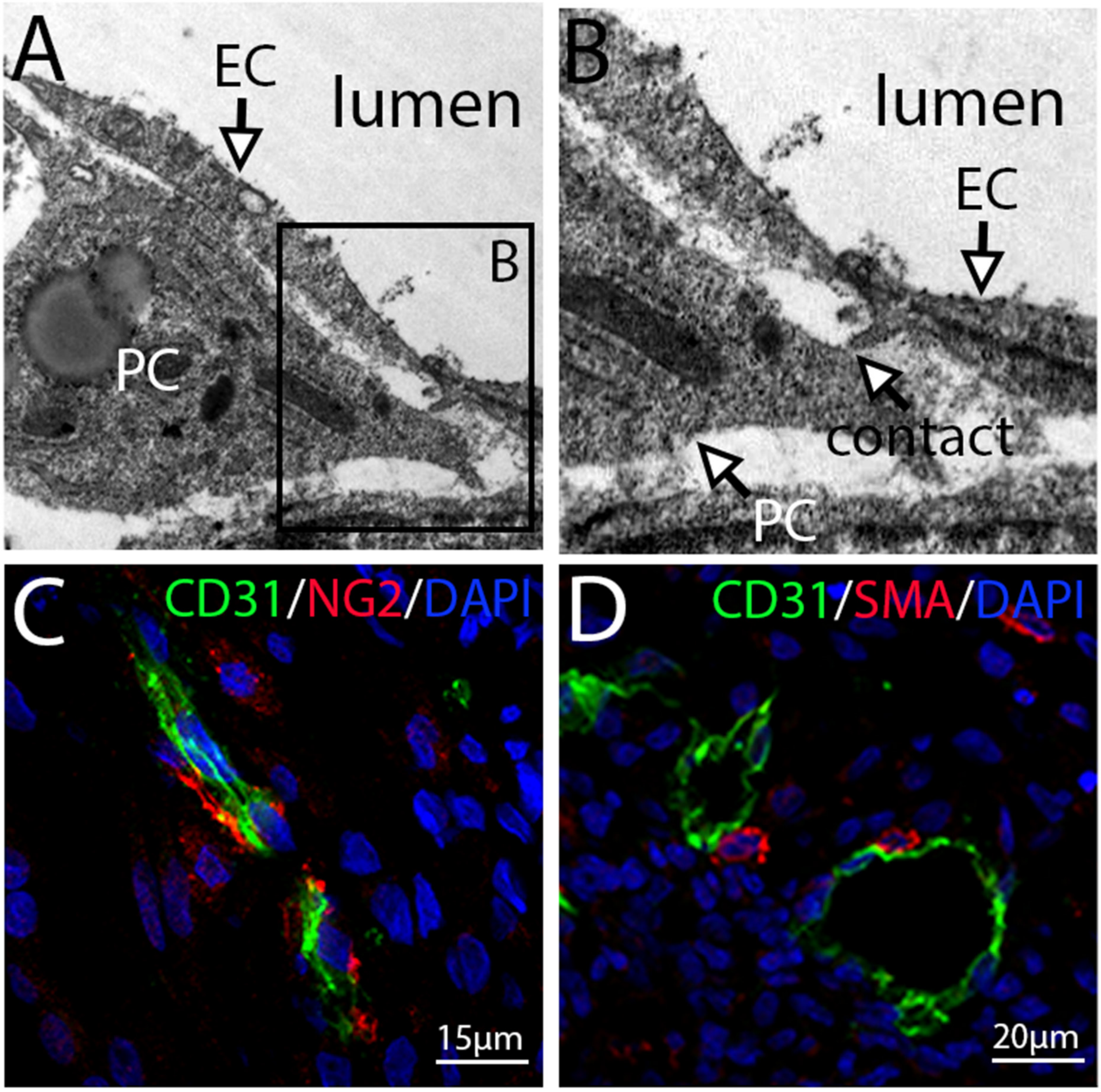

Figure S9: Ultrastructural analysis of endothelial/periendothelial cell interaction and pericyte staining.

(A-B) Endothelial cells (EC) and periendothelial cells (PC) show direct finger-like contact sites as similarly observed in pericyte-endothelial cell or myoendothelial interactions *in vivo*. B shows a higher magnification of A. (C-D) A part of the periendothelial cells are $NG2^{+}$ and α-SMA^{+}.

Figure S10

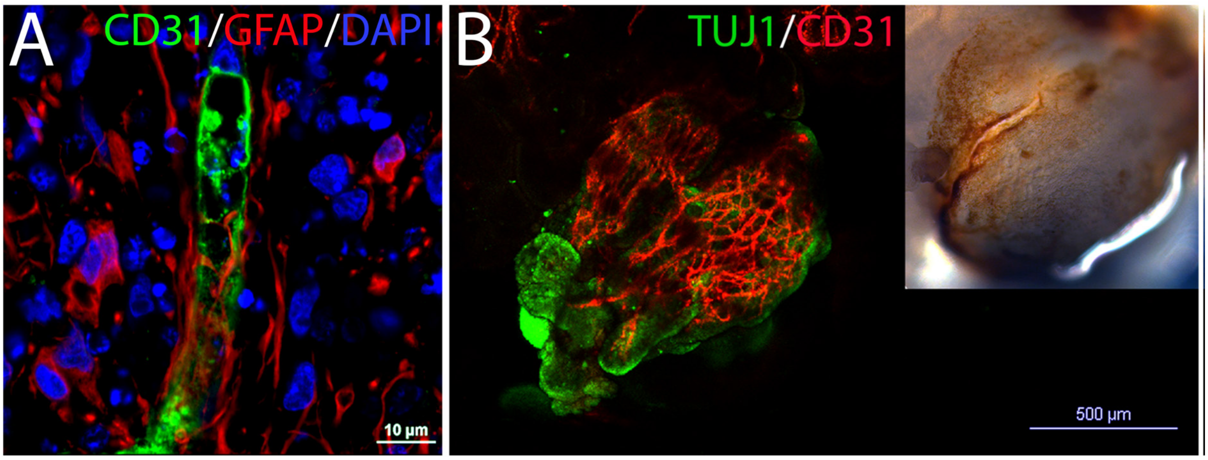

Figure S10: CD31⁺ vessels within the neural part of the organoids.
(A) CD31⁺ vessel-like structures invade the neural part of the organoid being closely associated with GFAP⁺ cellular processes. (B) The formation of an extensive vascular network can be found at sites of pigmented epithelium formation.

Figure S11

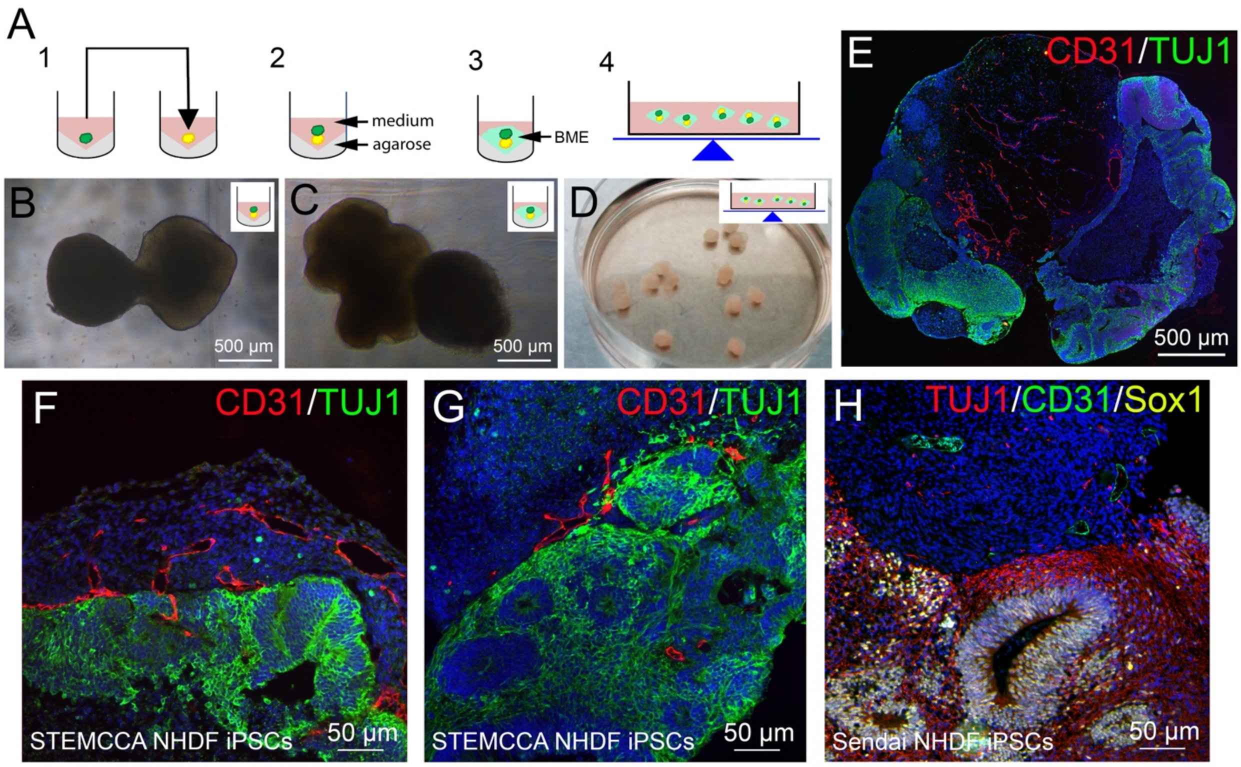

Figure S11: An improved protocol for the generation of vascularized neural organoids.

(A) Schematic representation of the workflow. (1) Mesodermal and neural aggregates of defined size are generated within single wells of an agarose coated 96-well plate. (2) A neural aggregate is transferred to a well already containing a mesodermal aggregate and both are co-cultured. (3) Attached aggregates are embedded into basement membrane extract (BME). (4) Embedded aggregates are transferred to a 6-well plate and cultured on a rocking table in the humidified incubator. (B) Neural and mesodermal aggregate attached to each other forming a dumbbell-like structure. (C) Fused aggregates embedded in BME. (D) Vascularized neural organoids generated from STEMCCA NHDF iPSCs 30 days after BME embedding. (E-G) Immunofluorescence analyses of vascularized neural organoids at day 30 after BME embedding. E shows an overview about the whole organoid, revealing $CD31^{+}$ vascularized mesodermal parts (red) and $TUJ1^{+}$ neural parts (green). Higher magnifications are depicted in F-G. (H) A vascularized neural organoid from Sendai NHDF iPSCs is shown.

Movie S1: Distribution of the endothelial network within tumor organoids.

The movie shows the distribution of the $CD31^+$ endothelial network (red) within a tumor organoid. Tumor cells are GFP^+ (green). Images were obtained by confocal laser scanning microscopy of a whole mount stained tumor organoid. The organoid morphology is flattened due to squashing of the whole-mount under the cover slip. Vessel-like structures can be observed around a core of GFP-positive tumor cells. Some endothelial sprouts are found penetrating the tumor cell mass.

Movie S2: Blood flow towards tumor organoid.

A tumor organoid (green) transplanted to the chorioallantoic membrane of a chicken embryo is shown at day 3 after transplantation.

Movie S3: Vascular network within the mesodermal part of a neural organoid.

The vascular network within the mesodermal part of the neural organoid is visualized using immunofluorescence analyses with a specific antibody targeted against CD31. A whole mount staining was performed and the tissue was subsequently cleared using ethylcinnamate. The video shows different planes of a confocal z-stack.
